# Self-patterning Gd nano-fibers in Mg-Gd alloys

**DOI:** 10.1038/srep38537

**Published:** 2016-12-07

**Authors:** Yangxin Li, Jian Wang, Kaiguo Chen, Meiyue Shao, Yao Shen, Li Jin, Guo-zhen Zhu

**Affiliations:** 1 State Key Laboratory of Metal Matrix Composites, Shanghai Jiao Tong University, 800 Dongchuan Rd., Shanghai, 200240, P.R. China; 2 National Engineering Research Center of Light Alloy Net Forming, School of Materials Science and Engineering, Shanghai Jiao Tong University, 800 Dongchuan Rd., Shanghai, 200240, P.R. China; 3 Department of Mechanical and Materials Engineering, University of Nebraska-Lincoln, Lincoln, NE, 68588, USA; 4 National Key Lab of Shockwave and Detonation Physics, Institute of Fluid Physics, China Academy of Engineering Physics, Mianyang, Sichuan, 621000, P.R. China

## Abstract

Manipulating the shape and distribution of strengthening units, e.g. particles, fibers, and precipitates, in a bulk metal, has been a widely applied strategy of tailoring their mechanical properties. Here, we report self-assembled patterns of Gd nano-fibers in Mg-Gd alloys for the purpose of improving their strength and deformability. 1-nm Gd nano-fibers, with a 〈c〉-rod shape, are formed and hexagonally patterned in association with Gd segregations along dislocations that nucleated during hot extrusion. Such Gd-fiber patterns are able to regulate the relative activities of slips and twinning, as a result, overcome the inherent limitations in strength and ductility of Mg alloys. This nano-fiber patterning approach could be an effective method to engineer hexagonal metals.

Microstructure engineering is a persistently vigorous technique in altering material’s properties through tailoring geometrical features of structural units at multiple length scales and modifying three-dimensional arrangements of structural units. Structural units can be classified into three, two, one, or zero dimensions, such as three-dimensional volumes^1^,^2^ (phases, grains, particles/precipitates), two-dimensional surfaces^3^,^4^ (boundaries, interphases), one-dimensional lines (triple lines, edges, dislocations) or zero-dimensional points (quadruple points, vertices on polyhedral particles)^5^. In advancing the material’s performance resulted from these structural units, arranging their distribution provides different strategies in addition to regulating the dimensions and shapes of these structural units. To realize specific microstructure, heat treatment, mechanical deformation, and/or their combinations can be applied while adjusting chemical compositions of a material.

Rare-earth (RE) elements^6^ has a demonstrated significance in tuning the microstructure of Mg alloys, such as weakening basal texture of Mg alloys^7^,^8^, refining grain sizes, forming long-period stacking-ordered structure^4^, etc. RE solutes can also be trapped in twin boundaries, and in turn, impeding migration of twin boundaries^9^,^10^. In addition, the elastic interactions between solutes and dislocations lead to solute segregation and depletion around dislocation cores. Such a solute atmosphere, which can maintain their confined structural and chemical states over a range of evaluated temperatures^11^, produces a drag force on the moving dislocations, pins the dislocation motion, and thus modifies the mechanical performance of materials.

Here, we reported self-assembled hexagonal patterns of Gd-segregated dislocations that have a 〈c〉-rod shape. These pinned dislocations act as the predictable inhibitor for basal slips because the glide of basal dislocations must cut them, but less affect the glide of non-basal prismatic dislocations because they are on the parallel planes. Additionally, the crystal domains, strengthened by such pinned dislocation patterns, can effectively impede twin propagation. As a result, such dislocation patterns can change the relative mobility of plastic deformation carriers (dislocations and twins).

## Methods

Mg-1Gd (wt.%) alloy billets were prepared through melting high purity Mg (99.99%) and Mg-25 wt.%Gd master alloys in an electric furnace under a protective gas mixture of SF_6_/CO_2_. The billets were partly indirect extruded, at temperatures of 400 °C with extrusion ratio of 16, up to 150 mm from the die. After that, the die and butt were removed from the machine and quenched together into a water bath. Samples then were isochronally annealed at 200 °C, 250 °C, 300 °C for 2–4 hours. TEM samples were prepared through twin-jet electro-polishing methods and additional ion polishing at 500 eV for 0.5 h using Gatan precision ion polishing system (PIPS II MODEL 695). Structural characterization was carried out under scanning transmission electron microscopy (STEM) mode at 200 kV using a JEOL-ARM200F microscope with a probe-forming lens corrector. Due to the large difference in atomic numbers of Gd and Mg, high-angle annular dark-field (HAADF) imaging technique was applied to image Gd atoms in binary Mg-Gd alloys.

## Results

**Figure 1:**
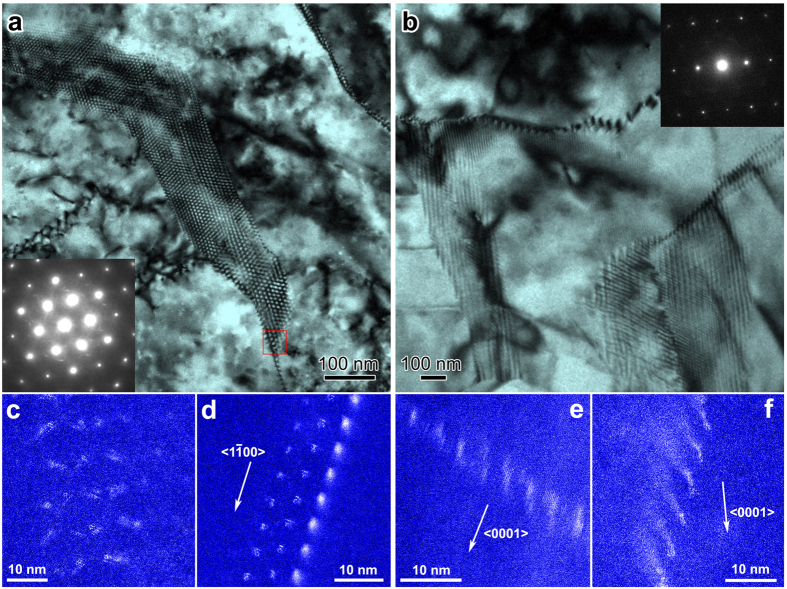
Gd nano-fiber patterns. (**a** and **b)** are Transmission Electron Microscopy Bright-Field (TEM-BF) images of the typical Gd segregation patterns within binary Mg-Gd alloys, viewed from 〈0001〉 and 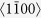, respectively. (**c**–**f**) scanning TEM High-Angle Annular Dark-Field (STEM-HAADF) images showing Gd segregation in the bright contrast. Panel c is the enlarged view of boxed region in (**a)**. (**d)** is a different Gd nano-fiber pattern viewed from 〈0001〉. The beam direction for (**e** and **f**) is 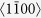.

Within binary Mg-Gd alloys after hot extrusion at 400 °C, we for the first time observed hexagonally patterned domains, which have a width of less than 200 nm and several microns in length, dispersive in the Mg matrix. Viewed from the 〈c〉-axis in Fig. 1a (more details in Fig. S1), each hexagonal pattern has an identical interspacing, which varies from 5 nm to 20 nm. Most domains have an interspacing of ~10 nm. The hexagonal patterns consist of Gd segregated clusters, shown as the bright intensity in the Z-contrast images (scanning transmission electron microscopy-high-angle annular dark-field, STEM-HAADF images) in Fig. 1c–f. The Gd segregations can slightly deviate from ideal hexagonal positions (see Fig. 1c,d). Viewed from the 〈b〉-axis (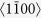) in Fig. 1b, straight-line features associated with the patterned fibers are characterized with interspacings of 5–15 nm. These straight-line features, lying along the 〈c〉-axis, turn out as Gd-rich fibers with ~1 nm in diameter. (Fig. 1e,f and Fig. S4). Combining the two orthogonal projections, we believe that Gd nano-fibers with the 〈c〉-rod shape are self-assembled into hexagonal patterns within the Mg matrix.

**Figure 2:**
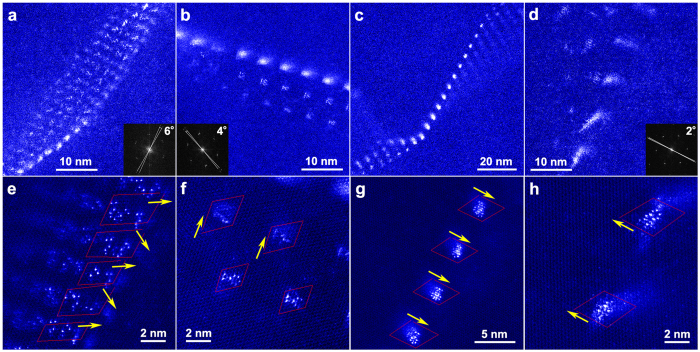
STEM-HAADF raw images of annealed samples. From left to right, Gd nano-fibers have an interspacing of ~3 nm, ~6 nm, and ~10 nm, in corresponding to a measurable rotation angle of 6°, 4°, and 2° between the adjacent lattices. Insets in (**a**,**b** and **d)** are the corresponding Fast Fourier Transform (FFT) images. (**e**–**h**) are enlarged view of regions selected from (**a**–**d**), respectively. The identified Burgers vectors, along crystallographic directions of 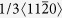, are labeled by yellow arrows. The Gd hexagonal rings and zigzag patterns within the dislocation cores are clearly recorded.

These directional nano-rods cannot exist without the dislocation module because the capillarity effect prevents them from forming at the nanoscale. Further crystallographic analysis reveals that these Gd nano-fibers are Gd-segregated dislocations. Some of these dislocations are characterized with the Burgers vector 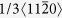 that is identified using the Burgers circuit method (see Fig. 2e–h). It is worth mentioning that short-range orders of Gd atoms, such as hexagonal rings and zigzag patterns, were recorded in more than 70% of the Gd segregations (see Figs 1c,d and 2). These characteristics of Gd short-range orders agree well with the observation in experiments and simulations^12^,^13^. The Gd rings always present at the dilated part of the dislocations because Gd has ~20% larger radius than Mg. The hexagonal patterns contain a set of Gd-segregated 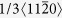 dislocations, which have the Burgers vectors either parallel or with 60° rotation, as shown in Fig. 2e,f. Some Gd-segregated dislocations may have Burgers vectors of 〈0001〉, because Burgers circuits are closed for such dislocations after we analyzed more than 20 hexagonal patterns (See those in Fig. 2f). The 〈c〉-screw character of these dislocations can be additionally supported by a large number of dislocations with 〈c〉-components through the Burgers vector analysis under two-beam conditions (See Fig. S3). These distinctive dislocations cause a few degrees misorientation between the adjacent grains bonded by one hexagonal pattern, as evidenced by sharp adjacent spots from the Fast Fourier Transform (FFT) of the hexagonal patterns (see insets in Fig. 2a,b and d).

The Gd nano-fiber hexagonal patterns are commonly observed near low-angle grain boundaries, which also consist of Gd-segregated dislocations (see details in Figs 1 and 2 and Fig. S2). A huge number of low-angle grain boundaries are randomly distributed within deformed grains with a few microns in size. The average spacing of Gd-segregated dislocations within the low-angle grain boundaries is around 3–15 nm, corresponding to the measurable misorientation angles of 1–6° about the 〈c〉-axis. This is in agreement with the analysis according to Frank’s law^14^. A significant Z-contrast of Gd nano-fiber patterns was detected when the interspacing of Gd-segregated dislocations is ~3–10 nm (see Fig. 2a–d). Gd-segregated dislocations are barely visible and irregularly patterned in a hexagonal shape as increasing the interspacing, e.g. 20 nm (see Fig. S5).

## Discussion

**Figure 3:**
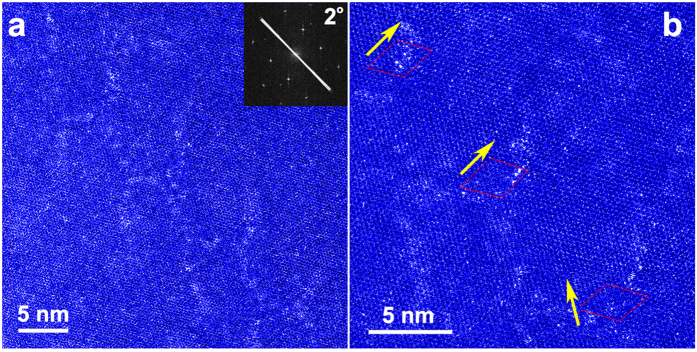
STEM-HAADF raw images of the as-extruded sample. The inset FFT graph in (**a**) shows that the adjacent lattices have a rotation angle of 2°. (**b**) enlarged view of the same region in (**a)**. Gd atoms, as bright dots in the HAADF images, has no clearly pattern but preferentially located near the defected regions. The identified components of Burgers vectors 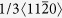 are labeled as yellow arrows.

Hexagonal patterns of Gd nano-fibers discovered in binary Mg-Gd alloys are dependent on annealing temperature. Before extrusion, Gd nano-fiber patterns were not detected in as-cast samples. The as-extruded microstructure was frozen by the indirect extrusion method, in which the die and butt were modified with fast quenching capability. Using TEM-BF techniques, we detected the same hexagonal patterns in as-extruded samples (Fig. S1) as in the annealed samples at 200 °C we showed before. The difference is that the as-extruded Gd segregation spreads in a “near-honeycomb” pattern (Fig. 3) or other irregular pattern (Fig. S6) with ~10 nm in diameter, compared to the two-dimensional arrays of Gd nano-fibers. An array of 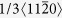 dislocations was observed with nearly random Gd solutes around their cores (Fig. 3b). These messy arrays further evolve into the perfect hexagonal pattern consisting of 1-nm Gd fibers after 200 °C and 250 °C annealing for 2–4 hours. We did not find any hexagonal pattern or 1-nm Gd fibers within the sample after 300 °C annealing (Fig. S7). The disappearance of such hexagonal pattern is possibly ascribed to recrystallization^15^. Thus, 1-nm Gd fibers and the corresponding hexagonal patterns would be favorably formed after moderate temperature annealing, (e.g. 200–250 °C) while destroyed at elevated temperatures.

**Figure 4:**
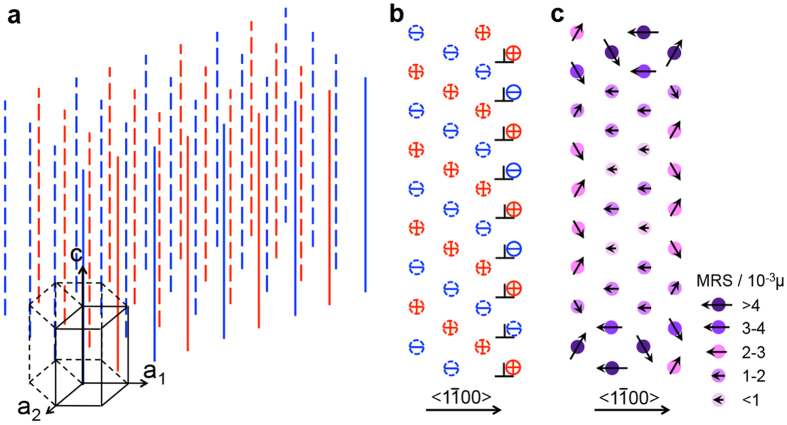
Schematics of the dislocations pattern. (**a**) 3D dislocation structure. The low-angle grain boundary is represented by an array of mixed 〈c + a〉 dislocations. 〈c〉 screw dislocations are represented in dashed lines, positive in red and negative in blue. The mixed dislocations are represented in solid lines, positive in red and negative in blue. Panel b shows the dislocations pattern viewed along 〈0001〉, and (**c**) is the distribution of maximum resolved stresses (MRS) on each dislocation.

The puzzle of patterning Gd fibers in a hexagonal shape can be understood in the framework of dislocation interaction. Corresponding to characteristics of dislocation patterns in Fig. 2, we assumed that a hexagonal pattern is comprised of 〈c〉 screw dislocations with alternative signs and an array of 〈c + a〉 dislocations, as shown in Fig. 4. This model was proposed based on the facts: (a) no extra misorientation was detected between the adjacent grains across the hexagonal pattern except the one caused by the low-angle grain boundary (represented by an array of 〈c + a〉 dislocations); (b) the FFT of the local area is very sharp indicating a sharp, rather than a gradual transition of orientations; (c) there is no elastic interactions between 〈c〉 screw dislocations and 〈a〉 edge dislocations because they are perpendicular to each other. We found that the maximum resolved stresses on individual slip systems are estimated to be smaller than 4 × 10^−3^ μ for the 〈c〉 screw dislocations inside the pattern as the interspacing is 10 nm. Where μ is shear modulus. Thus, the formation can be rationalized as follows. Once a low-angle grain boundary formed by the spatial pileup of 〈c + a〉 prismatic dislocations, 〈c〉 dislocations can come from two sources, either activated 〈c〉 slips or the resultant of 〈c + a〉 dislocations that are repelled by the grain boundary and react to each other and form 〈c〉 dislocations. These dislocations are then self-assembled in a hexagonal pattern due to the elastic interaction. Such patterns could be stabilized by the Peiers stress of the 〈c〉 screw dislocation, which is greater than the maximum resolved stresses^16^,^17^.

From mechanical viewpoint, the pinned dislocations in the pattern could inhibit the easy basal slips by the “forest” strengthening mechanism^18^, for the purpose of strengthening Mg alloys, while crystal domains strengthened by such pinned dislocation patterns can effectively impede twin propagation, in turn, reducing twinning while improving deformability of Mg alloys. The tensile tests for normally extruded samples (containing no Gd-fiber pattern) and indirect-extruded samples (containing the embryo of Gd-fiber patterns) indicate that the self-patterning Gd nano-fibers do improve the mechanical responses (Fig. S9). Thus, introducing patterned Gd nano-fibers might provide a new path for manufacturing advanced hexagonal alloys in general.

## Additional Information

**How to cite this article**: Li, Y. *et al*. Self-patterning Gd nano-fibers in Mg-Gd alloys. *Sci. Rep.* **6**, 38537; doi: 10.1038/srep38537 (2016).

**Publisher’s note:** Springer Nature remains neutral with regard to jurisdictional claims in published maps and institutional affiliations.

## Supplementary Material

Supplementary Materals
