# Supplementary material for: Self-patterning Gd nano-fibers in Mg-Gd alloys
Source: Sci Rep. 2016 Dec 7;6:38537. doi: 10.1038/srep38537 (PMC5141485; doi:10.1038/srep38537)
Supplement: Supplementary Materals [file srep38537-s1.pdf]

Supplementary Material

Self-patterning Gd nano-fibers in Mg-Gd alloys

Yangxin Li [1,2] , Jian Wang [3] , Kaiguo Chen [4] , Meiyue Shao [2] , Yao Shen [1]* , Li Jin [2]* , Guo-zhen Zhu [1]*

[1]State Key Laboratory of Metal Matrix Composites, [2]National Engineering Research Center of Light Alloy Net Forming, School of Materials Science and Engineering, Shanghai Jiao Tong University, 800 Dongchuan Rd., Shanghai 200240, P.R. China.

[3]Department of Mechanical and Materials Engineering, University of Nebraska-Lincoln, Lincoln, NE, 68588, USA.

[4]National Key Lab of Shockwave and Detonation Physics, Institute of Fluid Physics, China Academy of Engineering Physics, Mianyang, Sichuan 621000, P.R. China.

[1] Correspondence and requests for materials should be addressed to Y.S. (Email: yaoshen@sjtu.edu.cn), L.J. (Email: j_jinli@sjtu.edu.cn), or G.-z. Z. (zhugz@sjtu.edu.cn).

Experimental Methods:

1. Experimental alloys

Mg-1Gd (wt.%) alloy billets, with 60 mm in diameter and 50 mm in length, were prepared through melting high purity Mg (99.99%) and Mg-25wt.%Gd master alloys in an electric furnace under a protective gas mixture of SF_6/CO_2. The billets were partly indirect extruded at temperatures of 400ºC with extrusion ratio of 16. The billets were heated to the extrusion temperature and held isothermally for 30 mins before extrusion. Extrusion experiments were stopped when the billets had been extruded to 150 mm from the die, and the die and butt were removed from the machine and quenched together into a water bath. As the extrusion butt was quenched immediately after extrusion, static recrystallization was hindered and the dynamically recrystallized microstructure during extrusion was preserved. After extrusion, samples were isochronally annealed at 200°C, 250°C, 300°C for 2-4 hours.

2. Sample preparation and TEM characterization

The as-fabricated Gd-Mg alloys were cut into slices with ~600 μm in thickness, and then mechanically ground to ~100 μm. TEM discs with 3 mm in diameter were punched out from the slices, and then twin-jet electro-polished in an ethanol solution with 4 pct perchloric acid. TEM specimens were further thinned at 500 eV for 0.5 h using Gatan precision ion polishing system (PIPS II MODEL 695). Structural characterization was carried out under scanning transmission electron microscopy (STEM) mode at 200 kV using a JEOL-ARM200F microscope with a

probe-forming lens corrector. Due to the large difference in atomic numbers of Gd and Mg, high-angle annular dark-field (HAADF) imaging technique was applied to image Gd atoms in binary Mg-Gd alloys. The probe convergence semi-angle was approximately 30 mrad. The collection semi-angle of the annular dark-field (ADF) detector was ~68-280 mrad.

Supplementary Text:

1. Distribution of the hexagonal patterns

Viewed along [0001], Figure S1 shows a typical grain of a few microns in size. Since hot extrusion was carried out at 400°C and cooled down immediately, no twins were detected in as-extruded samples and those with 200°C and 250°C annealing. Even in the as-extruded sample in Figure S1, we did not find a high density of dislocations, except some grains with dislocation pile-up near their grain boundaries. At grain boundaries, Gd segregation with short-range ordering was clearly observed, which is consistent with the previous report.[(1)] Within most grains, we detected a huge number of low-angle grain boundaries, consisting of segregated dislocations. Some of these low-angle grain boundaries accompanies with hexagonal patterns, labeled as the red arrows in Figure S1a. Those hexagonal patterns are less than 200 nm in width and a few microns in length. The interspacing within those hexagonal patterns is usually in the range of 3-20 nm, with a predominant interspacing of ~10 nm.

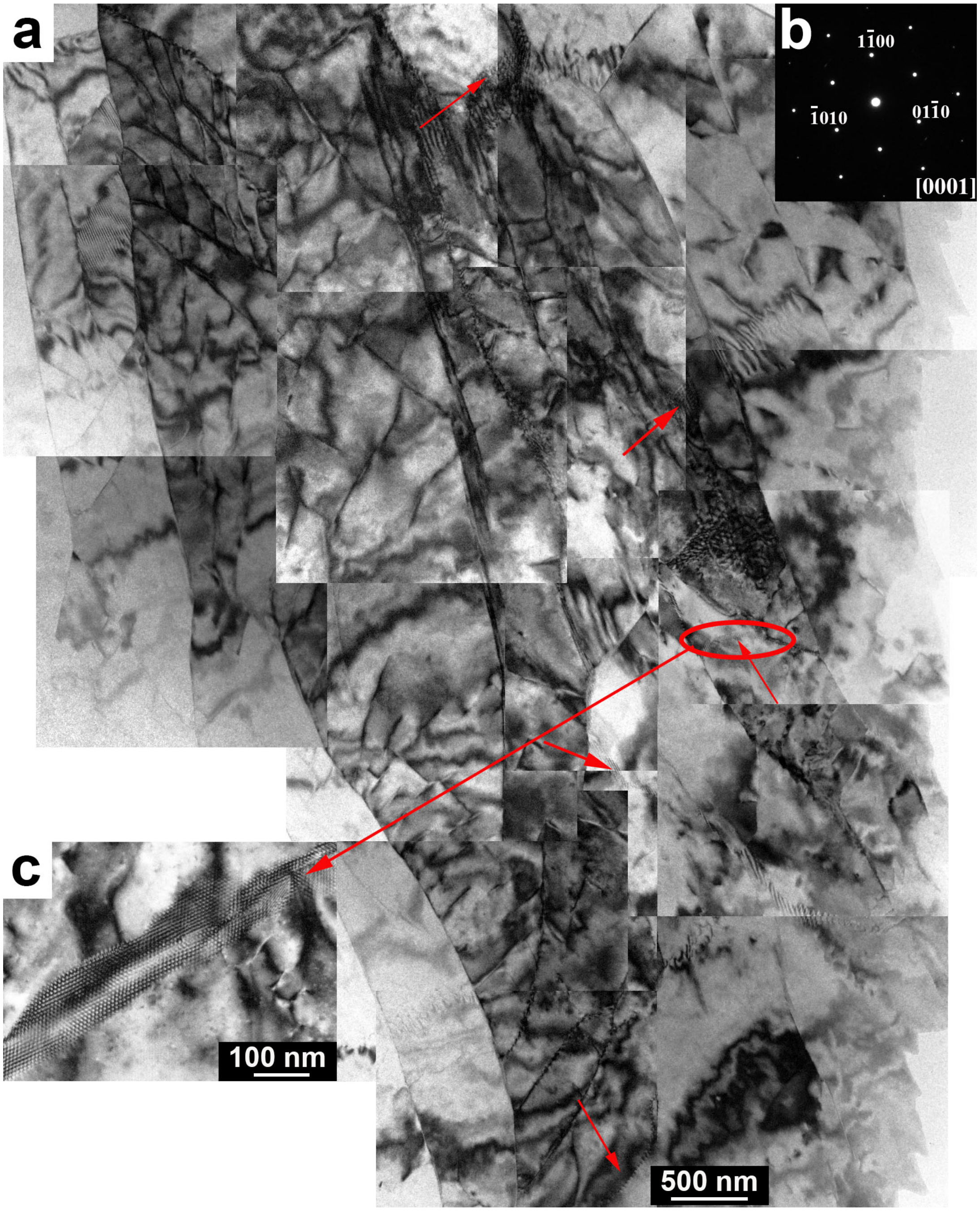

Figure S1. TEM images showing the distribution of hexagonal patterns in one grain viewed along the [0001], as indicated from the diffraction pattern in **b**. **c**, An enlargement of the elliptical area in **a**. Such hexagonal patterns, with <200nm in width and ~ micron in length, are always connected with low-angle grain boundaries.

2. Low-angle grain boundaries with Gd-segregated dislocations

When the electron beam is along <0001>, low-angle grain boundaries consist with a set of Gd-segregated dislocations, showing as the array of bright dots in the HAADF images in Figure S2. Most low-angle grain boundaries detected are along $<1\bar{1}00>$. The associated dislocations have the identified Burgers vector of $\frac{1}{3}<11\bar{2}0>$. The identified Burgers vectors can change their crystallographic directions, e.g. with 60° rotation, corresponding to the steps and kinks at the low-angle grain boundaries. Some low-angle grain boundaries are along $<11\bar{2}0>$, with alternating dislocations of identified Burgers vector $\frac{1}{3}<11\bar{2}0>$. It should be noted that we couldn't identify the Burgers vector for a few low-angle grain boundaries, because the dislocations in these boundaries show a pure <c> component, parallel to the beam direction. As shown in the red arrows in Figure S2, the hexagonal patterns of Gd segregation were found to form along low-angle grain boundaries. In addition, the hexagonal patterns are preferentially located beside Gd-segregated dislocations changing their Burgers vector directions.

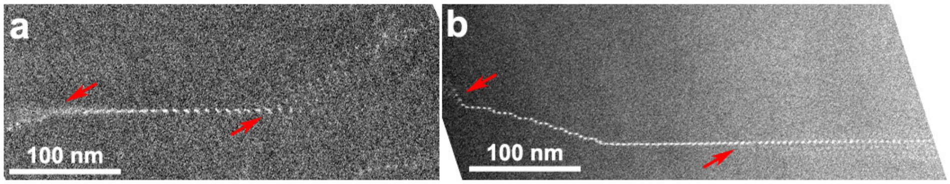

Figure S2. STEM-HAADF raw images of Gd-segregated arrays. The low-angle grain boundaries were always found at the start or end of these Gd segregated arrays. Most low-angle grain boundaries are along $<1\bar{1}00>$ and those arrays preferentially start with the dislocations changing their identified Burgers vectors (also see Figure 2).

3. Burgers vector with c-components

The types of dislocations were identified under different two-beam conditions, g = 0002 and g = $10\bar{1}0$, respectively. The invisible dislocations in Figure S3b with diffraction vector of g = $10\bar{1}0$, have Burgers vectors with <c>-component. It should be noted that cross-slip trails were observed, suggesting the activation of <c> slips. The existence of Burgers vectors <0001> and the activation of <c> slips are required to form the hexagonal patterns.

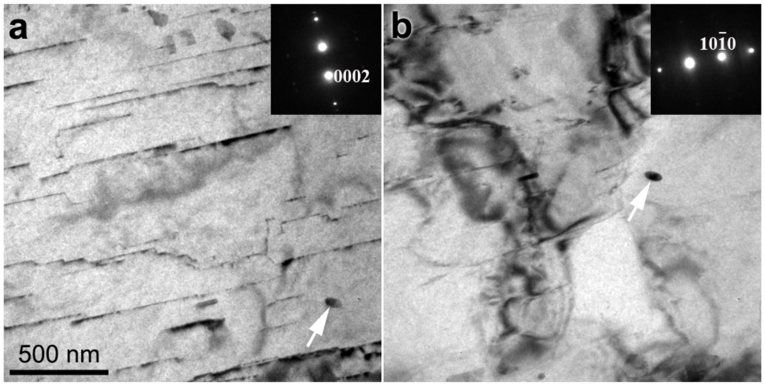

Figure S3. TEM-BF images under two-beam conditions with diffraction vector of g=0002 (**a**) and g=$10\bar{1}0$ (**b**), respectively. The dislocation with <c>-components is confirmed. The white arrows labeled the same area in **a** and **b**.

4. Gd-segregation viewed from $<1\bar{1}00>$

Figure S4 shows the hexagonal patterns when the electron beam is along $<1\bar{1}00>$. These Gd-nano-fibers are along <0001>. As shown in Figure S4b, the interspacing between the segregated lines is approximately 5 nm.

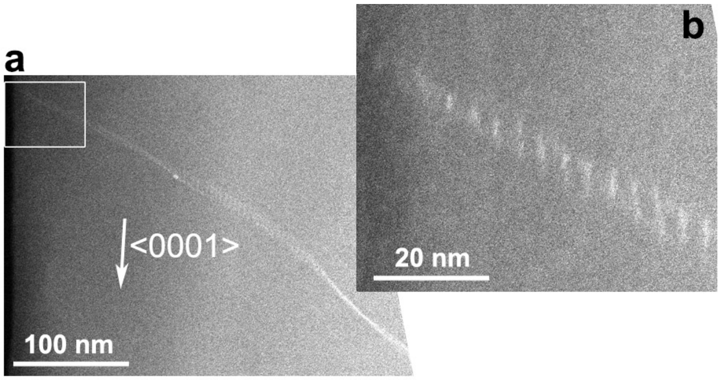

Figure S4. STEM-HAADF raw images showing Gd nano-fibers viewed from $<1\bar{1}00>$. **b**, Enlarged view of the boxed region labeled in **a**.

5. Hexagonal pattern with large interspacing

Viewed along <0001>, some hexagonal patterns have a large interspacing, e.g. 20 nm, compared to the typical value of 10 nm. (See Figure S5a). As shown in Figure S5b and c with enhanced contrast after Fast Fourier Transform (FFT) filtering, those hexagonal patterns include barely visible Gd-segregations with a cellular structure. Due to its weak contrast, it is hard to locate the template, the low-angle grain boundary with 20 nm interspacing between dislocations.

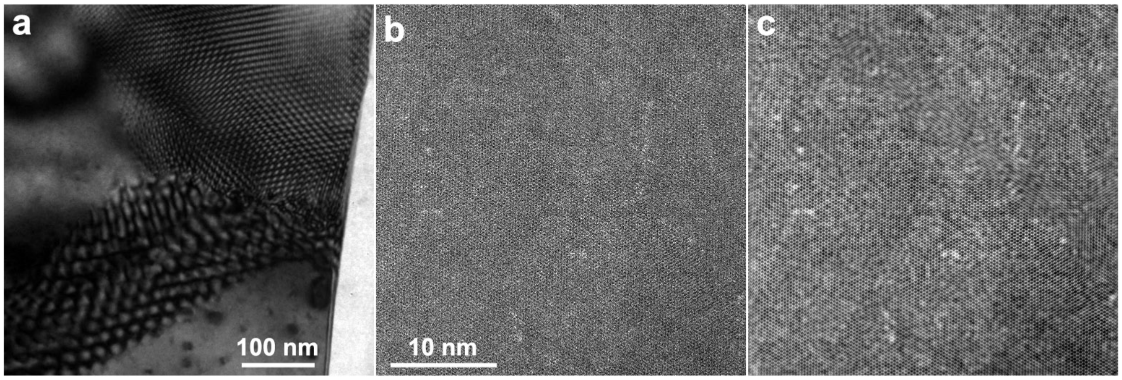

Figure S5. The hexagonal pattern showing in TEM-BF images in **a** with interspacing of ~20 nm. **b**, The STEM-HAADF raw images of hexagonal pattern with ~20 nm interspacing. **c**, The FFT filtered image of **b** .

6. Hexagonal pattern in as-excluded samples

As shown in Fig S6, hexagonal patterns are under developed in as-excluded samples. Viewed along <0001>, Burgers vectors of $\frac{1}{3}<11\bar{2}0>$ were identified at some Gd-segregations. The cellular structure has relatively weak contrast because Gd solutes have not been fully segregated into dislocations.

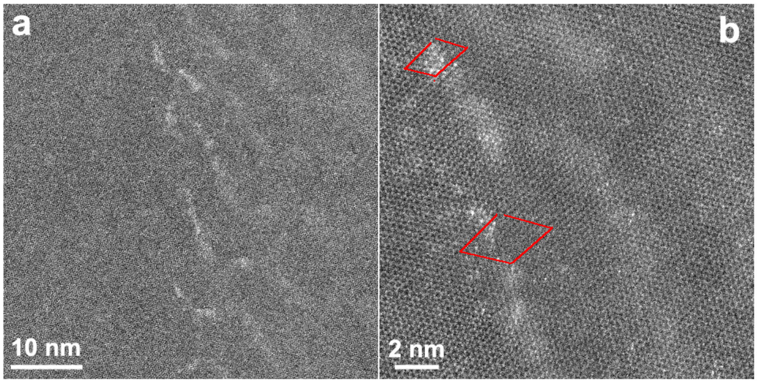

Figure S6. STEM-HAADF raw images of as-excluded sample indicating the evolution of Gd segregated arrays. **b**, Enlarged view of **a**.

7. Microstructure after 300°C annealing

Gd-segregations and their hexagonal patterns form right after hot extrusion, have better shape after 200°C and 250°C annealing, and can be destroyed at higher temperature. Within samples after 300°C annealing (see TEM images in Fig. S7), annealing twins, instead of hexagonal patterns, were recorded. We did not find twins in as-extruded samples and annealed samples at 200°C and 250°C. The existence of annealing twins is consistent with the fact that Mg alloys has a recrystallization temperature of ~260°C *(15)*.

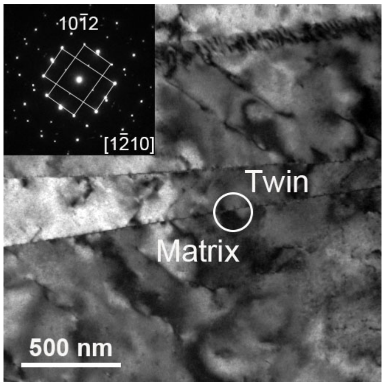

Figure S7. TEM-BF images of annealed sample at 300°C for 4 hours, showing the formation of an annealing twin.

8. Hexagonal pattern of dislocations

Figure S8 is schematics of the dislocation components in the patterned fibers. The columns other than the one forming the low-angle grain boundary are composed of screw dislocations of alternative signs, and the grain boundary dislocations are of <a+c> dislocations, with the signs of <a> edge dislocations identical but those of the <c> screw dislocations alternating. Such a picture comes up with several facts: (1) There is no contribution of misorientation across these fibers from these non-grain boundary dislocations, as the misorientaion of the parts outside of the

fibers are the same of that produced by the single column of low-angle grain boundary dislocations; (2) The diffraction pattern of the local area is very sharp indicating a sharp, rather than gradual, transition of orientations; (3) There is no interactions between screw dislocations and edge dislocations since it is isotropic in the basal plane of a hexagonal lattice and isotropic theory works exactly for dislocations parallel to the <c>-axis; (4) Gd fibers are formed by segregation to dislocation cores.

Stability of such patterns is evaluated by checking the maximum resolved stress on each slip system of the non-grain-boundary screw dislocations. The grain boundary are stabilized by its edge components and its screw components are thus anchored to the edge ones. Simple calculations show that when the number of dislocations in the column is larger than the order of 10, the maximum resolved stress on a screw dislocation from all other dislocation in the pattern are below 4×10^{-3} μ except for the one or two dislocations at each end. These columns can be stabilized since the Peiers stress of the dislocations, the minimum stress needed to move a straight dislocation, is well above 4×10^{-3} μ for the dislocations in the prismatic planes with the pinning effect of Gd solution *(16-17)*. The dislocations at each end can be stabilized by other defects such as grain boundary or threading dislocations.

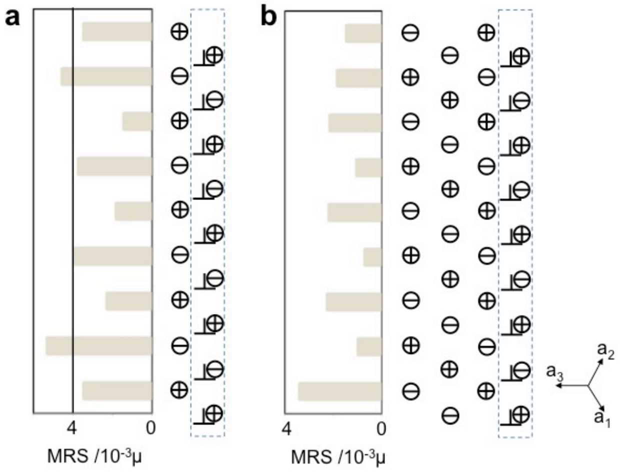

Figure S8. Schematics of the dislocations patterns. **a**, A pattern of two columns, **b**, A pattern of multiple (four) columns. The low-angle grain boundaries are composed of mixed <c+a> dislocations, while other columns are of screw (<c>, in circles) dislocations. A circle with "+" inside denotes a positive screw dislocation, while one with "-" inside a negative screw dislocation. a_1, a_2 and a_3 show the $<11\bar{2}0>$ directions in the basal plane. The bar-figures show the maximum resolved stress (MRS) of the left or left-most column on one of the three <a> slip systems.

8. Tensile Responses

Due to these difficulties in quantifying the volume ratio of Gd-fiber patterns, we cannot quantify the Gd fiber-reinforced effect on the mechanical properties. A few tensile tests indicate the positive effect caused by the Gd-fiber pattern. In order to maintain the identified texture caused from hot extrusion, we compared the tensile results of extruded samples and indirect-extruded samples. The indirect-extruded samples, with the embryo of Gd-fiber patterns, have at least 20% strengthen effect compared to the normally extruded samples, which have no Gd-fiber pattern.

The normally extruded samples experience additional annealing treatment associated to the normal extrusion processes, which destroys any possible Gd-fiber pattern. In addition, we performed the tensile tests for indirect-extruded samples, with Gd-fiber patterns after additional aging at 200°C. As shown in Figure S9, the self-patterning Gd-fibers do slightly increase both the strength and ductility even after intermediate annealing treatment.

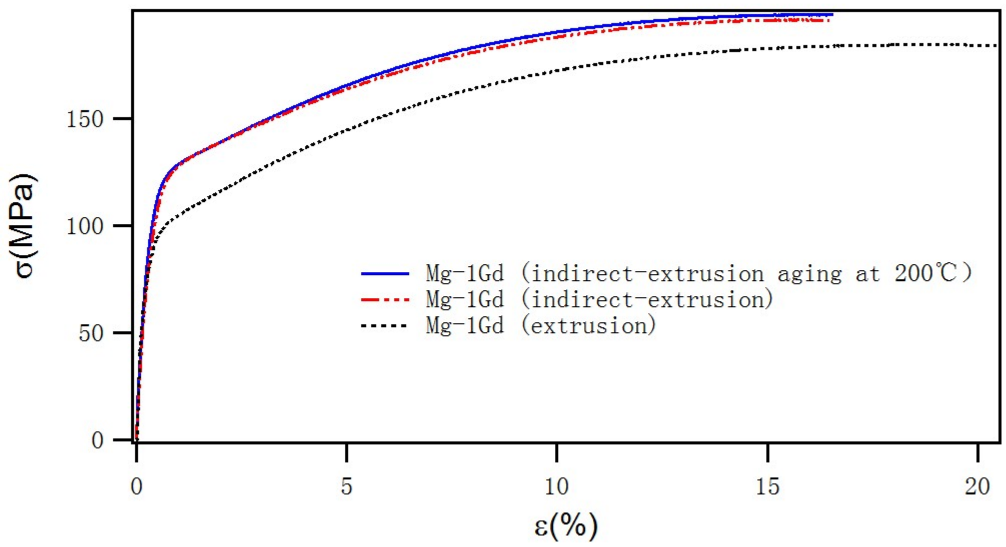

Figure S9. Tensile results of Mg-Gd alloys with/without Gd nano-fiber patterns. The black, red and blue curves present the mechanical responses of Mg-Gd alloys without any patterns, with the embryo, and with the Gd-fiber patterns, respectively.

References

1. Bugnet, M. et al. Segregation and clustering of solutes at grain boundaries in Mg-rare earth solid solutions. Acta Mater. 79, 66–93 (2014).
